# Thrombogenesis-associated genetic determinants as predictors of thromboembolism and prognosis in cervical cancer

**DOI:** 10.1038/s41598-023-36161-w

**Published:** 2023-06-12

**Authors:** Beatriz Vieira Neto, Valéria Tavares, José Brito da Silva, Joana Liz-Pimenta, Inês Soares Marques, Luísa Carvalho, Lurdes Salgado, Deolinda Pereira, Rui Medeiros

**Affiliations:** 1 Molecular Oncology and Viral Pathology Group, Research Center of IPO Porto (CI-IPOP)/ Pathology and Laboratory Medicine Dep., Clinical Pathology SV/ RISE@CI-IPOP (Health Research Network), Portuguese Oncology Institute of Porto (IPO Porto) / Porto Comprehensive Cancer Centre (Porto.CCC), 4200-072 Porto, Portugal; 2 FMUP, Faculty of Medicine, University of Porto, 4200-072 Porto, Portugal; 3 ICBAS, Abel Salazar Institute for the Biomedical Sciences, Rua de Jorge Viterbo Ferreira, 228, 4050-313 Porto, Portugal; 4 Oncology Department, Portuguese Institute of Oncology of Porto (IPOP), 4200-072 Porto, Portugal; 5 Department of Medical Oncology, Centro Hospitalar de Trás-os-Montes e Alto Douro (CHTMAD), 5000-508 Vila Real, Portugal; 6 FCUP, Faculty of Sciences, University of Porto, 4169-007 Porto, Portugal; 7 External Radiotherapy Department, Portuguese Institute of Oncology of Porto (IPOP), 4200-072 Porto, Portugal; 8 Research Department, Portuguese League Against Cancer (NRNorte), 4200-172 Porto, Portugal; 9 CEBIMED, Faculty of Health Sciences, Fernando Pessoa University, 4200-150 Porto, Portugal

**Keywords:** Cancer, Cell biology, Genetics, Molecular biology, Biomarkers, Cardiology, Diseases, Gastroenterology, Molecular medicine, Oncology

## Abstract

Venous thromboembolism (VTE) is a leading cause of death among cancer patients. Khorana score (KS) is the most studied tool to predict cancer-related VTE, however, it exerts poor sensitivity. Several single-nucleotide polymorphisms (SNPs) have been associated with VTE risk in the general population, but whether they are predictors of cancer-related VTE is a matter of discussion. Compared to other solid tumours, little is known about VTE in the setting of cervical cancer (CC) and whether thrombogenesis-related polymorphisms could be valuable biomarkers in patients with this neoplasia. This study aims to analyse the effect of VTE occurrence on the prognosis of CC patients, explore the predictive capability of KS and the impact of thrombogenesis-related polymorphisms on CC-related VTE incidence and patients’ prognosis regardless of VTE. A profile of eight SNPs was evaluated. A retrospective hospital-based cohort study was conducted with 400 CC patients under chemoradiotherapy. SNP genotyping was carried on by using TaqMan® Allelic Discrimination methodology. Time to VTE occurrence and overall survival were the two measures of clinical outcome evaluated. The results indicated that VTE occurrence (8.5%) had a significant impact on the patient’s survival (log-rank test, *P* < 0.001). KS showed poor performance (KS ≥ 3, χ^2^, *P* = 0.191). *PROCR* rs10747514 and *RGS7* rs2502448 were significantly associated with the risk of CC-related VTE development (*P* = 0.021 and *P* = 0.006, respectively) and represented valuable prognostic biomarkers regardless of VTE (*P* = 0.004 and *P* = 0.010, respectively). Thus, thrombogenesis-related genetic polymorphisms may constitute valuable biomarkers among CC patients allowing a more personalized clinical intervention.

## Introduction

Thrombosis is a common and often deadly cardiovascular disease that arises from the generation of a thrombus in the arterial or venous circulation, often followed by the formation of an embolus (a foreign or abnormal particle circulating in the blood), which can lead to the reduction or even blockage of the blood flow, a condition known as thromboembolism^1^. Venous thromboembolism (VTE) encompasses deep venous thrombosis (DVT), its most common manifestation, and pulmonary embolism (PE), a consequence of DVT in most scenarios (~ 95%)^2–5^. Like other common and complex diseases, VTE pathophysiology involves both inherited and acquired factors^2–4^. The disease is thought to have a strong genetic component, with over 50% of the variation of VTE risk being attributed to inherited factors^6^. In the last decades, several VTE-related genetic markers have been discovered in the general population, most of them located in the vicinity or within haemostatic genes, as expected. These genetic factors are known to downregulate anticlotting mechanisms and, in opposition, upregulate proclotting ones^1,3^. The list includes mutations, particularly in genes related to the anticoagulation system (*PROC*, *PROS1* and *SERPINC1*), but mainly genetic polymorphisms, which have a higher relevance at a population level given their high frequency (≥ 1%)^4^. The most studied genetic polymorphisms underlying VTE susceptibility are the single-nucleotide polymorphisms (SNPs) *F5* rs6025 (Factor V Leiden (FVL)) and *F2* rs1799963 (prothrombin G20210A), which are associated with activated protein C (APC) resistance and increased expression levels of prothrombin, respectively, both favouring thrombogenesis^7–9^. As for the acquired risk factors, the more impacting ones are advanced age, high body mass index (BMI), autoimmune disorders and cancer^1^.

Malignancy is an independent risk factor for VTE development. The association between cancer and VTE was first observed by Trousseau in the nineteenth century^10^. Cancer-related VTE, or the Trousseau syndrome as it is known, affects over 15% of oncological patients and constitutes the second most frequent cause of death among them^4,11–15^. Beyond the disease itself, its detrimental effect on the prognosis of cancer patients is thought to be also attributed to the contribution of the deregulated haemostatic mechanisms that underlie the disease pathogenesis, in both cancer progression and aggressiveness^4,16,17^. Therefore, identifying tools able to predict cancer-related VTE is demanding as it may allow a more tailored prophylaxis. Additionally, it is extremely important to unravel new predictive biomarkers because cancer patients are more prone to bleeding events, particularly under anticoagulation therapy. In clinical practice, the most studied VTE risk-assessment model is the Khorana score (KS), which encompasses the parameters of tumour site, pre-treatment BMI, platelet, leukocyte and haemoglobin counts, to categorise cancer patients according to VTE risk^18,19^. Unfortunately, the score performance is poor and the search for predictive biomarkers of cancer-related VTE is still ongoing^20–24^. Whether genetic determinants related to thrombogenesis in the general population could predict cancer-related VTE is a matter of discussion^4,24^.

In general, cancer-related VTE is attributed to factors related to the patient (e.g., advanced age), tumour (e.g., primary site, histology and stage) and antineoplastic treatments (e.g., surgery and chemotherapy), which affect blood composition, blood flow and the integrity of blood vessels (Virchow’s Triad)^1,16^. Among solid tumours, gastric and pancreatic cancers are associated with the highest VTE risk, while gynaecological tumours are associated with an intermediate risk, with prostate, breast and skin cancers, among others, constituting the low-risk group^24^. Compared with other gynaecological cancers, such as ovarian and endometrial tumours, the data concerning VTE incidence and its effect on cervical cancer (CC) patients as well as the underlying mechanisms, are not as solidified. According to the scarce data, VTE incidence in these patients appears to be substantial, which is in part attributed to the disease therapeutic management^25–27^. The standard treatment for CC patients at locally advanced disease stages (IB2 to IVA) consists of concomitant chemoradiotherapy (CCRT), which exposes these patients to an increased risk of thrombotic events^26,28–30^. In this context, the present study was designed to analyse the incidence of VTE following CC diagnosis and its impact on the outcome of patients with this malignancy from the Northern Region of Portugal, treated with CCRT. Furthermore, the predictive ability of KS was investigated, as well as the predictive and prognostic value (regardless of VTE status) of thrombogenesis-related genetic polymorphisms previously identified in the general population.

## Materials and methods

### Study cohort description

A retrospective hospital-based cohort study was conducted with cytological and histologically diagnosed CC patients with European ancestry, admitted to the Clinic of Gynaecology of the Portuguese Institute of Oncology of Porto (IPOP), from February 2002 to October 2009 and from May 2017 to October 2021, for the first-line treatment, from whom biological samples were available in our biobank. Patients were included if they had disease staged as IB2 to IVA and were submitted to CCRT as the first line treatment, with weekly administration of 40 mg/m^2^ of cisplatin during external radiotherapy, and regarding on response followed or not by brachytherapy. Patients were excluded if they were under 18 years old, had done surgery (a known VTE risk factor) at least 6 months before CCRT or were only admitted for a second opinion. A total of 400 CC patients from the north region of Portugal were enrolled. Tumour staging was made according to the International Federation of Gynaecology and Obstetrics (FIGO) staging system^31^.

Patients were classified as having a VTE history in the setting of cancer based on their medical files. No active screening for VTE was made since this measure is not included in the clinical routine procedures at IPOP. As such, there was no accountability for clinically asymptomatic events, which represent the majority of cancer-related VTE events and are also considered to negatively impact a patient’s prognosis, although to a lesser extent^32,33^. Data concerning demographic and clinicopathological factors, as well as the follow-up of the patients, were also obtained from their medical files. The characterization of the study cohort (n = 400) according to the VTE status (with VTE vs. without clinical evidence of VTE) is given in Table 1. The mean follow-up was 155.3 months (standard deviation (SD) = 7.6 months).

**Table 1:** Demographic and clinicopathological characteristics of CC patients according to VTE status (n = 400).

Variable (no. patients)*	VTE status			*P*-value
	With VTE (n = 34; 8.5%)	Without clinical evidence of VTE (n = 366; 91.5%)	Total (n = 400)	
Age (400)				
Median	50.0	48.0	49.0	–
≥ 50 years**	17 (50.0)	164 (44.8)	181	0.688
< 50 years**	17 (50.0)	202 (55.2)	219	
Missing data	–	–	–	
Cancer stage (394)				
I and II	22 (64.7)	276 (76.7)	298	0.179
III and IV	12 (35.3)	84 (23.3)	96	
Missing data	–	6	6	
Cancer histology (393)				
Adenocarcinoma and others	8 (24.2)	63 (17.5)	71	0.467
Squamous cell carcinoma	25 (75.8)	297 (82.5)	322	
Missing data	1	6	7	
KS (90)				
< 2	6 (54.6)	49 (62.0)	55	0.883
≥ 2	5 (45.5)	30 (38.0)	35	
0	0 (0.0)	0 (0.0)	0	0.191
1–2	8 (72.7)	72 (91.1)	80	
≥ 3	3 (27.3)	7 (8.9)	10	
Missing data	23	287	310	

*CC* cervical cancer, *No.* number, *VTE* venous thromboembolism, *KS* Khorana score.

*Number of patients with available data.

**Cut-off defined based on the median age of the patients as the variable is not normally distributed (Kolmogorov–Smirnov test, *P* < 0.001).

This study was approved by the ethics committee at IPOP (CES IPO: 287A/014). A written consent according to the principles of the Helsinki Declaration was obtained from each patient before their enrolment in this study.

### Sample collection and genomic DNA extraction

Peripheral venous blood samples of each patient were obtained using a standard technique and collected in ethylenediaminetetraacetic acid (EDTA)-containing tubes.

From the blood samples, genomic Deoxyribonucleic Acid (DNA) was extracted using GRS Genomic DNA Kit—Blood & Cultured Cells from Grisp Research Solutions®, as indicated by the manufacturer’s instructions.

### Polymorphism selection

To cover different aspects of haemostasis, polymorphism selection was made considering genome-wide association studies (GWAS)-identified SNPs linked to VTE susceptibility in the general population, as well as SNPs associated with platelet activation and aggregation (primary haemostasis)^34–37^. From the identified variants, were prioritized those with reported prognostic value in the context of cancer independently of VTE status. In addition, a minor allele frequency (MAF) of 10% in the Iberian population (Ensembl database, https://www.ensembl.org/index.html; accessed on 20th September 2021) was considered to avoid missing SNP genotypes in the cohort study (n = 400). Following these criteria, eight SNPs were selected: *Protein C receptor* (*PROCR*) rs10747514, *Regulator of G Protein Signalling 7* (*RGS7*) rs2502448, *OTU Deubiquitinase 7A* (*OTUD7A*) rs7164569, *Zinc finger protein, FOG family member 2* (*ZFPM2*) rs4734879, *Integrin Subunit Beta 3* (*ITGB3*) rs5918, *Glutathione-Disulfide Reductase* (*GSR*) rs3779647, *Coagulation factor 11* (*F11*) rs4253417 and *Contactin 6* (*CNTN6*) rs6764623.

### Polymorphism genotyping

Polymorphism genotyping was conducted using the TaqMan® Allelic Discrimination methodology in a StepOne Plus Real-time Polymerase Chain Reaction (Real-time PCR) system (Applied Biosystems). Each reaction was performed using 2.5 µL of TaqPath™ ProAmp™ Master Mix (1×), 2.375 µL of sterile water, 0.125 µL of TaqMan® Genotyping Assay Mix (assays ID provided in Table 2) and 1.0 µL of genomic DNA, making a total volume of 6 µL. The thermal cycling conditions for DNA amplification were the following: (1) 95 °C for 10 min (activation of Taq DNA Polymerase), (2) 45 cycles of 95 °C for 15 s (denaturation of DNA chains), and (3) 60 °C for 1 min (primers pairing and extension). Data of DNA amplification were analysed using StepOne Software (version 2.3 Applied Biosystems). Negative controls (without DNA) were included in each RT-PCR reaction to prevent false positives. Moreover, to ensure the quality of SNP genotyping, a double sampling for at least 10% of randomly selected DNA samples was performed, with an accuracy of over 99%. Genotyping results were evaluated by two researchers with no previous knowledge concerning VTE status, demographic and clinicopathological data of the patients included in the study.

**Table 2:** Genotype distribution of thrombogenesis-related SNPs in a cohort of 400 CC patients.

SNP	MAFi (MA)*	Genotype	N (%)	N total (%)	MAFs (MA)	Failed genotyping (%)	TaqMan SNP genotyping assay ID
*PROCR* rs10747514	22% (A)	GG	131 (48.9)	268 (100)	30% (A)	132 (33.0)	C___1825060_10
		AG	112 (41.8)				
		AA	25 (9.3)				
*RGS7* rs2502448	38% (C)	TT	102 (40.0)	255 (100)	39% (C)	145 (36.3)	C__26887460_10
		CT	109 (42.7)				
		CC	44 (17.3)				
*OTUD7A* rs7164569	34% (G)	AA	122 (32.5)	375 (100)	45% (G)	25 (6.3)	C___1698935_20
		AG	165 (44.0)				
		GG	88 (23.5)				
*ZFPM2* rs4734879	34% (G)	AA	123 (45.7)	269 (100)	31% (G)	131 (32.8)	C___1315535_10
		AG	124 (46.1)				
		GG	22 (8.2)				
*ITGB3* rs5918	14% (C)	TT	205 (67.9)	302 (100)	18% (C)	98 (24.5)	C____818008_30
		CT	86 (28.5)				
		CC	11 (3.6)				
*GSR* rs3779647	47% (C)	TT	90 (27.8)	324 (100)	46% (C)	76 (19.0)	C__25472374_10
		CT	167 (51.5)				
		CC	67 (20.7)				
*F11* rs4253417	42% (C)	TT	96 (32.8)	293 (100)	43% (C)	107 (26.8)	C__32291269_10
		CT	141 (48.1)				
		CC	56 (19.1)				
*CNTN6* rs6764623	25% (C)	AA	143 (57.9)	247 (100)	24% (C)	153 (38.3)	C__26850683_10
		AC	89 (36.0)				
		CC	15 (6.1)				

*SNP* single-nucleotide polymorphism, *MAFi* minor allele frequency in the Iberian population, *MAFs* minor allele frequency in the study cohort, *MA* minor allele, *ID* identification number.

*According to the *Ensembl* database.

### Statistical analysis

Data analysis was carried on by using the computer software IBM® SPSS® Statistics software package Version: 28.0.0.0. (IBM Corp. Released 2021). Associations of the genetic variants with VTE status and patient’s demographic and clinicopathological factors were assessed using the chi-square test (χ^2^) for categorical variables, while the student’s t-test or the Mann–Whitney U test was employed for continuous variables depending on their distribution (normal and not normal distribution, respectively). The distribution was assessed using the Kolmogorov–Smirnov test given the large cohort size.

Two measures of clinical outcome were considered, namely time to VTE occurrence and overall survival (OS). The former was defined as the period from cancer diagnosis to VTE occurrence (primary endpoint) or patients’ last clinical assessment, while the latter was the time from cancer diagnosis to patient death from all causes (secondary endpoint) or last clinical assessment. Survival curves were obtained using the Kaplan–Meier method, while the probabilities of survival were analysed using the log-rank test. After an initial comparison between the survival curves under the additive genetic model, the most suitable genetic model (additive, recessive or dominant) for each genetic polymorphism was selected. In terms of survival, stratified analyses by VTE status (with vs. without clinical evidence of VTE), FIGO stage (III/IV vs. I/II) and age (< 50 vs. ≥ 50 years) were conducted for polymorphisms with at least a marginal association considering the entire cohort (*P* < 0.100). In addition to the log-rank test, univariate Cox regression analyses were also performed to estimate the hazard ratio (HR) values regarding the 10-year risk of VTE occurrence and the 10-year risk of death associated with the relevant genetic polymorphisms. Multivariate Cox regression analyses were also conducted adjusting for FIGO stage (III/IV vs. I/II) and age (< 50 vs. ≥ 50 years), which are known prognostic factors in CC^38,39^. All tests conducted were two-sided and a 5% level of significance was established.

### Ethics approval

This study was approved by the ethics committee at IPOP (CES IPO:287A/014). A written consent according to the principles of the Helsinki Declaration was obtained from each patient before their enrolment in this study.

## Results

### Genotype distribution of the thrombogenesis-related genetic variants

The genotype distribution of each selected SNP and the respective TaqMan assay are represented in Table 2. Overall, except for *RGS7* rs2502448, there were no significant statistical differences between the different genotypes of each variant and the patient’s demographic and clinicopathological characteristics (data not shown), namely FIGO stage (III/IV vs. I/II), age (< 50 years vs. ≥ 50 years) and histological subtype (adenocarcinoma/others vs. squamous cell carcinoma) (*P* > 0.05). The distribution of *RGS7* rs2502448 genotypes among the different histological subtypes was statistically different (χ^2^, *P* = 0.019). Namely, most patients with squamous cell carcinoma had the variant C allele (63.55%; n = 203), while most of those with other histological subtypes had the TT genotype (56.25%; n = 48). Nevertheless, in this study, cancer histology was not a predictor of CC-related VTE nor a prognostic factor (*P* > 0.05).

### Cancer-associated-VTE: impact of VTE on the clinical outcome of cervical cancer patients

**Figure 1:**
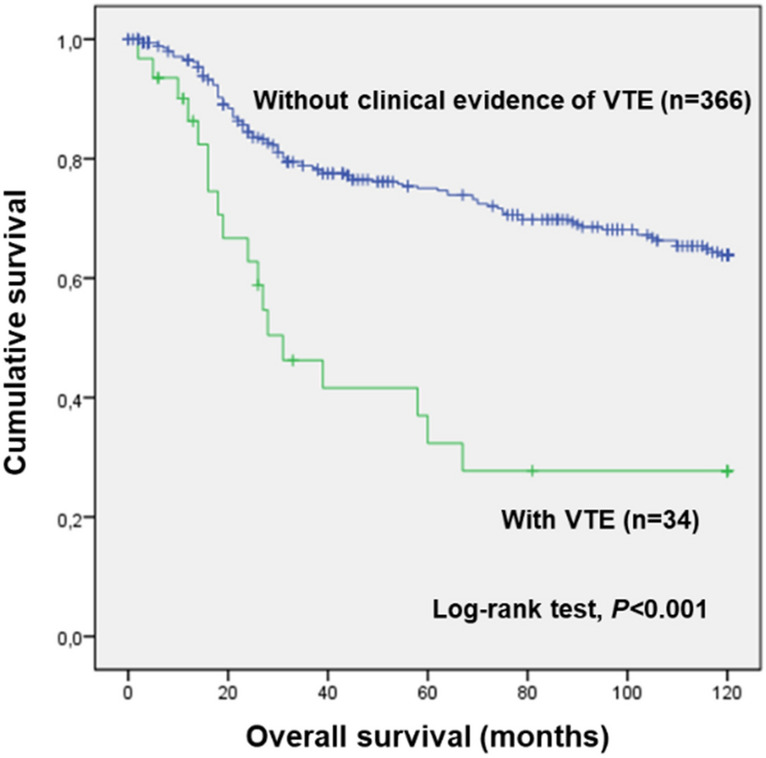
10-year overall survival (OS) by Kaplan–Meier and Log-rank test for CC patients (n = 400), according to VTE status. Patients with VTE had a lower 10-year OS compared to the ones without clinical evidence of VTE (*P* < 0.001). Specifically, VTE patients had a mean 10-year OS of 60.9 months (SD = 8.8 months), while their counterparts presented a mean 10-year OS of 92.3 months (SD = 2.3 months).

In the study, 34 patients (8.5%) presented symptomatic VTE, the mean time to the disease occurrence being 37.2 months after initiation of cancer management (SD = 52.5 months). VTE patients had a lower 10-year OS compared to the ones without clinical evidence of the disease (mean 10-year OS of 60.9 months (SD = 8.8 months) and 92.3 months (SD = 2.3 months), respectively; log-rank test, *P* < 0.001; Fig. 1). This result was corroborated by univariate Cox regression analysis (VTE vs. without clinical evidence of VTE; HR 2.522; 95% confidence interval (CI), 1.530–4.159; *P* < 0.001).

From the entire cohort, only 90 patients had the necessary data available for KS determination, (tumour site, pre-treatment platelet and leukocyte count, haemoglobin level and BMI). Following the original cut-off scores (KS ≥ 3: high risk; 1–2: intermediate risk; 0: low risk), 80 out of the 90 patients were considered as having intermediate risk and 10 were high risk (Table 1). VTE incidence was 10% and 30% among the former and the latter, respectively (χ^2^, *P* = 0.191). Considering the new recommended cut-off scores (KS ≥ 2, high risk; 0–1, low risk), 55 out of the 90 patients were considered as having low risk and 35 were high risk (Table 1). The disease frequency was 14% and 22% among the former and the latter, respectively (χ^2^, *P* = 0.883). Considering only the original cut-offs, which had better performance, KS was found to be only marginally associated with time to VTE occurrence (*P* = 0.078). Stratified analyses according to FIGO stage (III/IV vs. I/II) and patient’s age (< 50 vs. ≥ 50 years) also did not reveal a significant predictive impact on the score. Nevertheless, KS showed a prognostic value among the 90 patients (log-rank test, *P* = 0.046). Namely, patients with a score ≥ 3 had a lower 10-year OS compared to the ones with a score < 3 (mean 10-year OS of 33.4 months (SD = 5.9 months) and 48.2 months (SD = 2.0 months), respectively). As it was only possible to determine the score for 90 patients and given its poor performance, further analyses integrating the score were not conducted.

### Thrombogenesis-related genetic variants: impact on VTE occurrence and clinical outcome

Considering the entire cohort of CC patients, for *ZFPM2* rs4734879, *OTUD7A* rs7164569, *ITGB3* rs5918, *GSR* rs3779647, *F11* rs4253417 and *CNTN6* rs6764623, no statistically significant association with the time to VTE occurrence and patient’s prognosis was observed (*P* > 0.05). In opposition, *PROCR* rs10747514 and *RGS7* rs2502448 polymorphisms were shown to have a significant impact.

**Figure 2:**
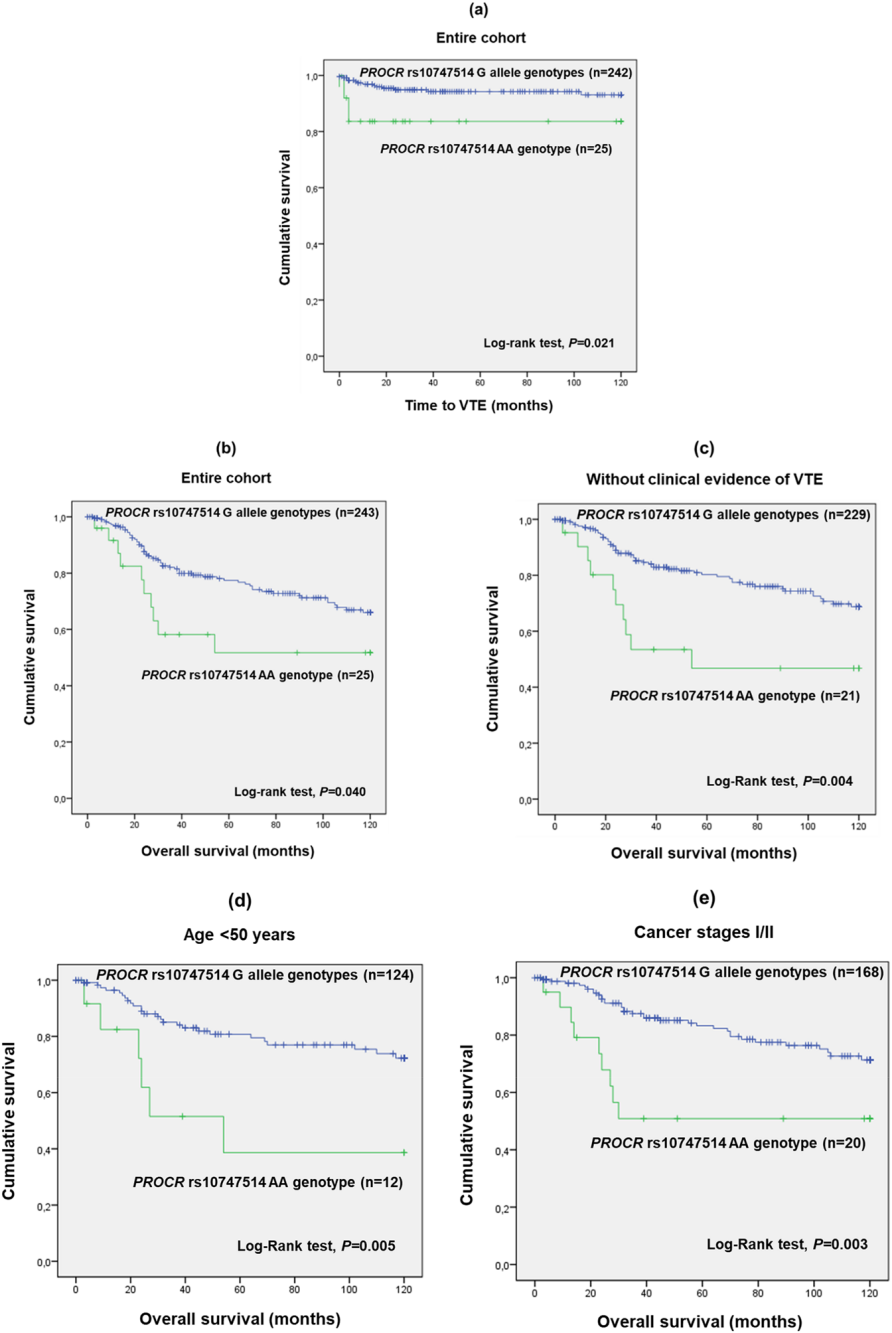
(**a**) Time to VTE occurrence by Kaplan–Meier and Log-rank test for CC patients, according to *PROCR* rs10747514 (n = 267). Patients with the AA genotype presented a lower time to VTE development compared to the G allele carriers (mean time to VTE of 113.7 months (SD = 8.8 months) and 100.8 months (SD = 1.7 months), respectively; log-rank test, *P* = 0.021). (**b**) 10-year overall survival (OS) by Kaplan–Meier and Log-rank test for CC patients, according to *PROCR* rs10747514 (n = 268). Patients with the AA genotype presented a lower 10-year OS compared to the G allele carriers (mean 10-year OS of 73.7 months (SD = 10. 8 months) and 95.3 months (SD = 2.8 months), respectively; log-rank test, *P* = 0.040). (**c**) 10-year overall survival (OS) by Kaplan–Meier and Log-rank test for CC patients without clinical evidence of VTE, according to *PROCR* rs10747514 genotypes (n = 250). Patients with the AA genotype had a lower 10-year OS compared to patients with the G allele (mean 10-year OS of 68.8 months (SD = 11.4 months) and 98.2 months (SD = 2.8 months), respectively; log-rank test, *P* = 0.004). (**d**) 10-year overall survival (OS) by Kaplan–Meier and Log-rank test for young CC patients (< 50 years) without clinical evidence of VTE, according to *PROCR* rs10747514 genotypes (n = 136). Patients with the AA genotype had a lower 10-year OS compared to patients with the G allele (mean 10-year OS of 62.1 (SD = 15.4 months) and 99.2 (SD = 3.8 months) respectively; log-rank test, *P* = 0.005). (**e**) 10-year overall survival (OS) by Kaplan–Meier and Log-rank test for CC patients at FIGO I/II stages and without clinical evidence of VTE, according to *PROCR* rs10747514 genotypes (n = 188). Patients with the AA genotype had a lower 10-year OS compared to carriers of G allele genotypes (mean OS of 70.6 months (SD = 11.9 months) and 101.0 months (SD = 3.0 months), respectively; log-rank test, *P* = 0.003).

The polymorphism *PROCR* rs10747514 presented a marginal association with VTE development (AA vs. AG/GG; χ^2^, *P* = 0.101). When analysing the time to a VTE event, the patients with the AA genotype (A being the minor allele; Table 2) exhibited a mean time of 100.8 months (SD = 8.8 months), whereas the G allele carriers presented a mean time of 113.7 months (SD = 1.7 months) under a recessive genetic model (AA vs. AG/GG; log-rank test, *P* = 0.021; Fig. 2a). The prothrombotic effect of the AA genotype was confirmed by univariate Cox regression analysis (AA vs. AG/GG; HR 3.463; 95% CI 1.128–10.369; *P* = 0.030). Furthermore, the multivariate analysis also corroborated the predictive impact of rs10747514 (Table 3). Specifically, carriers of the AA genotype had a fourfold increase in the 10-year risk of VTE occurrence compared to G allele genotypes adjusted for FIGO stage (AA vs. AG/GG; adjusted HR (aHR) 4.273; 95% CI 1.342–13.601; *P* = 0.014; Table 3). The variant *PROCR* rs10747514 was also shown to have a potential prognostic value among CC patients (AA vs. AG/GG, log-rank test, *P* = 0.040; Fig. 2b). Specifically, patients with the AA genotype presented a mean 10-year OS of 73.7 months (SD = 10.8 months), whereas carriers of G allele genotypes exhibited 95.3 months (SD = 2.8 months), suggesting a detrimental impact of the AA genotype. By stratifying the analysis according to the patients’ VTE status (with vs. without clinical evidence of VTE), a significant impact of *PROCR* rs10747514 (Fig. 2c) on patients’ 10-year OS was observed among the group of patients without clinical evidence of VTE (log-rank test, *P* = 0.004; Fig. 2c) but not among those with VTE (AA vs. AG/GG; log-rank test, *P* = 0.087). In the group without clinical evidence of VTE, the carriers of the AA genotype had a lower 10-year OS compared to the G allele genotypes carriers, therefore corroborating a detrimental effect of the AA genotype (mean OS of 68.8 months (SD = 11.4 months) and 98.2 months (SD = 2.8 months), respectively; AA vs. AG/GG; log-rank test, *P* = 0.004; Fig. 2c). Considering solely the patients without clinical evidence of VTE, stratified analyses according to age (< 50 vs. ≥ 50 years) and FIGO stage (III/IV vs. I/II) were performed (Fig. 2d,e, respectively). The polymorphism was found to have a significant impact on the 10-year OS of only young patients (< 50 years) (AA vs. AG/GG; log-rank test, *P* = 0.005; Fig. 2d). In terms of the FIGO stage, a statistically significant impact on the 10-year OS was only observed for patients at FIGO I/II stages (AA vs. AG/GG; log-rank test, *P* = 0.003; Fig. 2e). In line with the previous results, univariate Cox regression analysis confirmed the prognostic value of *PROCR* rs10747514 among those without clinical evidence of VTE (AA vs. AG/GG; HR 2.578; 95% CI 1.308–5.083; *P* = 0.006). Furthermore, according to multivariate analysis, those with the AA genotype had also a threefold increase in the 10-year risk of death compared to G allele genotypes when adjusted for FIGO stage (AA vs. AG/GG; aHR 2.612; 95% CI 1.322–5.161; *P* = 0.006; Table 4).

**Table 3:** Multivariate analysis (backward Wald method) on the VTE risk of CC patients considering the polymorphisms *PROCR* rs10747514 (n = 262) and *RGS7* rs2502448 (n = 249).

Variable	Time to VTE					
	Initial model			Final model*		
	aHR	95% CI	*P*-value	aHR	95% CI	*P*-value
*PROCR* rs10747514 (AA vs. AG/GG^1^)	4.282	**1.335–13.737**	**0.014**	4.273	**1.342–13.601**	**0.014**
FIGO stage (III/IV vs. I/II^1^)	2.736	**1.005–7.447**	**0.049**	2.740	**1.011–7.426**	**0.048**
Age (< 50 vs. ≥ 50 years^1^)	1.001	0.961–1.042	0.975	–	–	–

Variable	Time to VTE					
	Initial model			Final model*		
	aHR	95% CI	*P*-value	aHR	95% CI	*P*-value
*RGS7* rs2502448 (CT/CC vs. TT^1^)	10.486	**1.384–79.433**	**0.023**	10.672	**1.409–80.813**	**0.022**
FIGO stage (III/IV vs. I/II^1^)	2.887	**1.072–7.777**	**0.036**	2.818	**1.048–7.579**	**0.040**
Age (< 50 vs. ≥ 50 years^1^)	1.468	0.532–4.048	0.459	–	–	–

*VTE* venous thromboembolism, *CC* cervical cancer, *aHR* adjusted hazard ratio, *CI* confidence interval.

^1^Reference group.

*After applying the backward Wald method.

Bold values were considered statistically significant.

**Table 4:** Multivariate analysis (backward Wald method) on the risk of death of CC patients considering *PROCR* rs10747514 (n = 228) genotypes and *RGS7* rs2502448 (n = 228) genotypes.

Variable	10-year risk of death					
	Initial model			Final model*		
	aHR	95% CI	*P*-value	aHR	95% CI	*P*-value
*PROCR* rs10747514 (AA vs. AG/GG^1^)	2.846	**1.421–5.703**	**0.003**	2.612	**1.322–5.161**	**0.006**
FIGO stage (III/IV vs. I/II^1^)	1.600	0.890–2.878	0.116	1.593	0.888–2.859	0.118
Age (< 50 vs. ≥ 50 years^1^)	0.998	0.976–1.020	0.866	–	–	–

Variable	10-year risk of death					
	Initial model			Final model*		
	aHR	95% CI	*P*-value	aHR	95% CI	*P*-value
*RGS7* rs2502448 (CT/CC vs. TT^1^)	0.496	**0.291–0.846**	**0.010**	0.490	**0.288–0.835**	**0.009**
FIGO stage (III/IV vs. I/II^1^)	1.166	0.622–2.185	0.631	–	–	–
Age (< 50 vs. ≥ 50 years^1^)	0.835	0.492–1.416	0.503	0.822	0.487–1.388	0.463

*VTE* venous thromboembolism, *CC* cervical cancer, *aHR* adjusted hazard ratio, *CI* confidence interval.

^1^Reference group.

*After applying the backward Wald method.

Bold values were considered statistically significant.

**Figure 3:**
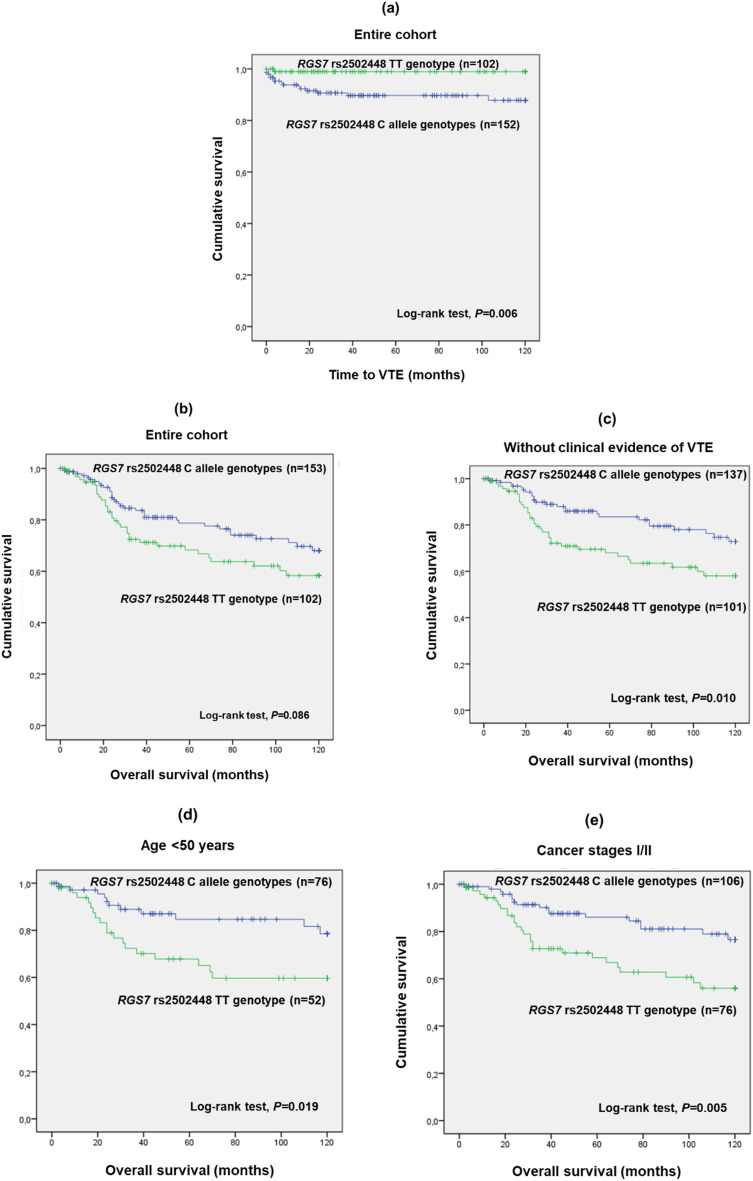
(**a**) Time to VTE occurrence by Kaplan–Meier and Log-rank test for CC patients, according to *RGS7* rs2502448 genotypes (n = 254). Patients with the C allele had a lower time to VTE occurrence compared to patients with the TT genotype (mean time to VTE of 118.8 months (SD = 2.9 months) and 108.5 months (SD = 1.2 months), respectively; log-rank test, *P* = 0.006). (**b**) 10-year overall survival (OS) by Kaplan–Meier and Log-rank test for CC patients, according to *RGS7* rs2502448 (n = 255). Patients with the C allele had a higher 10-year OS compared to patients with the TT genotype (mean 10-year OS of 97.1 months (SD = 3.5 months) and 86.0 months (SD = 4.9 months), respectively; log-rank test, *P* = 0.086). (**c**) 10-year overall survival (OS) by Kaplan–Meier and Log-rank test for CC patients without clinical evidence of VTE, according to *RGS7* rs2502448 genotypes (n = 238). Patients with the C allele genotypes had a higher 10-year OS compared to patients with the TT genotype (mean 10-year OS of 102.2 months (SD = 3.4 months) and 85.7 months (SD = 4. 9 months), respectively; log-rank test, *P* = 0.010). (**d**) 10-year overall survival (OS) by Kaplan–Meier and Log-rank test for young CC patients (< 50 years) and without clinical evidence of VTE, according to *RGS7* rs2502448 genotypes (n = 128). Patients with the C allele had a higher 10-year OS compared to TT genotype carriers (mean OS of 105.2 months (SD = 4.4 months) and 84.4 months (SD = 6.7 months), respectively; log-rank test, *P* = 0.019). (**e**) 10-year overall survival (OS) by Kaplan–Meier and Log-rank test for CC patients at FIGO I/II disease stages and without clinical evidence of VTE, according to *RGS7* rs2502448 genotypes (n = 182). Patients with the C allele genotypes had a higher 10-year OS compared to patients with the TT genotype (mean 10-year OS of 104.7 months (SD = 3.6 months) and 85.9 months (SD = 5.4 months), respectively; log-rank test, *P* = 0.005).

The polymorphism *RGS7* rs2502448 presented a significant association with VTE development (CT/CC vs. TT; χ^2^, *P* = 0.009). The patients with the C allele (minor allele; Table 2) presented a mean time to VTE of 108.5 months (SD = 2.9 months), contrasting with the 118.8 months (SD = 1.2 months) exhibited by TT genotype carriers under a dominant genetic model (CT/CC vs. TT; log-rank test, *P* = 0.006; Fig. 3a). This result suggested a prothrombotic effect of the C allele, which was corroborated by univariate Cox regression analysis (CT/CC vs. TT; HR 10.062; 95% CI 1.329–76.179; *P* = 0.025). Likewise, multivariate analysis showed that the patients with C allele genotypes had a tenfold increase in the 10-year risk of VTE occurrence compared to the TT genotype adjusted for FIGO stage (CT/CC vs. TT; aHR 10.672; 95% CI 1.409–80.813; *P* = 0.022; Table 3). The variant *RGS7* rs2502448 was also shown to have a potential prognostic value among CC patients given the marginal association (CT/CC vs. TT; log-rank test, *P* = 0.086; Fig. 3b). Specifically, C allele carriers presented a mean 10-year OS of 97.1 months (SD = 3.5 months), while the ones with the TT genotype exhibited a mean OS of 86.0 months (SD = 4.9 months), indicating a protective effect of the C allele. By stratifying the analysis according to the patients’ VTE status, a significant impact of the variant (Fig. 3c) on patients’ 10-year OS was observed only among those without clinical evidence of VTE (log-rank test, *P* = 0.010; Fig. 3c). In this group, carriers of the C allele had a higher 10-year OS compared to patients with the TT genotype, confirming the protective effect of the C allele (mean OS of 102.2 months (SD = 3.4 months) and 85.7 months (SD = 4.9 months), respectively; CT/CC vs. TT; log-rank test, *P* = 0.010; Fig. 3c).

Considering solely those without clinical evidence of VTE, stratified analyses according to age (< 50 vs. ≥ 50 years) and FIGO stage (III/IV vs. I/II) were performed (Fig. 3d,e, respectively). The polymorphism rs2502448 was found to have a statistically significant impact on the 10-year OS of only young patients (CT/CC vs. TT; log-rank test, *P* = 0.019; Fig. 3d). Additionally, regarding the FIGO stage, a significant impact of rs2502448 on the 10-year OS was only observed for patients at FIGO I/II stages (CT/CC vs. TT; log-rank test, *P* = 0.005; Fig. 3e). In line with the previous results, univariate Cox regression analysis confirmed the prognostic value of *RGS7* rs2502448 among the patients without clinical evidence of VTE. Specifically, C allele carriers had a 50% reduction in the 10-year risk of death compared to the ones with the TT genotype (CT/CC vs. TT; HR 0.513; 95% CI 0.305–0.862; *P* = 0.012). Likewise, according to multivariate analysis, the C allele was also associated with a 50% reduction in the 10-year risk of death compared to the TT genotype when adjusted for FIGO stage (CT/CC vs. TT; aHR 0.490; 95% CI 0.288–0.835; *P* = 0.009; Table 4).

Given the observed predictive and prognostic value of *PROCR* rs10747514 and *RGS7* rs2502448, multivariate analyses considering both polymorphisms were conducted adjusted for FIGO stage (III/IV vs. I/II) and age (< 50 vs. ≥ 50 years) (Table 5). The two polymorphisms had an addictive impact on CC-related VTE and patient survival.

**Table 5:** Multivariate analysis (backward Wald method) on the 10-year risk of VTE (n = 237) and the 10-year risk of death (n = 217) of CC patients.

Variable	Initial model			Final model*			Event
	aHR	95% CI	*P*-value	aHR	95% CI	*P*-value	
*PROCR* rs10747514 (AA vs. AG/GG^1^)	3.994	**1.112–14.342**	**0.034**	3.852	**1.071–13.860**	**0.039**	**10-year risk of VTE**
*RGS7* rs2502448 (CT/CC vs. TT^1^)	9.884	**1.299–75.212**	**0.027**	9.818	**1.289–74.746**	**0.027**	
FIGO stage (III/IV vs. I/II^1^)	3.200	**1.153–8.880**	**0.025**	3.223	**1.156–8.989**	**0.025**	
Age (< 50 vs. ≥ 50 years^1^)	1.418	0.503–4.003	0.509	–	–	–	
*PROCR* rs10747514 (AA vs. AG/GG^1^)	3.830	**1.752–8.373**	**0.001**	3.628	**1.683–7.822**	**0.001**	**10-year risk of death**
*RGS7* rs2502448 (TT vs. CT/CC^1^)	2.349	**1.331–4.147**	**0.003**	2.370	**1.346–4.171**	**0.003**	
FIGO stage (III/IV vs. I/II^1^)	1.367	0.719–2.599	0.341	–	–	–	
Age (< 50 vs. ≥ 50 years^1^)	0.992	0.570–1.724	0.977	–	–	–	

*VTE* venous thromboembolism, *CC* cervical cancer, *aHR* adjusted hazard ratio, *CI* confidence interval.

^1^Reference group.

*After applying the backward Wald method.

Bold values were considered statistically significant.

## Discussion

Cancer is a well-known major risk factor for VTE. A close interplay between tumour cells and the haemostatic components has been well-recognized, with thrombogenesis paralleling tumour progression and aggressive behaviour^1,2,25,40^. In concordance, beyond being highly incident, VTE imposes a negative impact on the prognosis of oncological patients^24^. As previously mentioned, data concerning VTE incidence and effect in CC patients, as well as the underlying mechanisms, is not as solidified^25–27^. Based on the somewhat scarce evidence, the frequency of venous thrombogenesis in these patients can reach up to 30% depending on multiple factors, including the type of therapeutic intervention^25^. Nevertheless, even in VTE absence, cancer patients often present a blood hypercoagulation state, which reflects the deregulated activity of certain haemostatic components involved in coagulation activation. As such, regardless of VTE status, haemostatic components present in the tumour microenvironment might favour cervical tumorigenesis, consequently deteriorating the patients’ prognosis^25,27,35^.

In this cohort study, 34 patients (8.5% of 400) presented clinical evidence of VTE in the setting of malignancy. However, as patients were not actively assessed for VTE presence, events among the ones without clinical evidence of the disease cannot be excluded. Nevertheless, the VTE incidence reported in the present study is comparable to the ones stated in the literature for CC patients under chemoradiotherapy (3%^41^, 4%^42^, 6%^43^, 8%^44^ and 15%^30^). According to the results, VTE patients had a lower 10-year OS compared to their counterparts (log-rank test, *P* < 0.001). More specifically, those with VTE presented almost a threefold increase in the risk of death compared to the ones without clinical evidence of the disease (HR 2.522; 95% CI 1.530–4.159; *P* < 0.001), suggesting that VTE significantly deteriorates CC patients’ prognosis (log-rank test, *P* < 0.001). Indeed, as previously stated, thromboembolism is known to negatively affect the prognosis of cancer patients. Furthermore, beyond the disease itself, tumour cells are known to hijack haemostatic components present in the surrounding microenvironment to fuel their progression towards metastasis, meanwhile disturbing the haemostatic imbalance towards thrombogenesis. Given the negative impact of VTE on the prognosis of CC patients, better thromboprophylaxis measures need to be explored, which also demands an improvement of VTE risk prediction models.

The most studied tool for the prediction of cancer-related VTE is KS^18,19^. In this study, the score had poor performance. While KS ≥ 2 was not found to be associated with increased VTE risk (χ^2^, *P* = 0.883), KS ≥ 3 was only marginally associated with the disease (χ^2^, *P* = 0.191) and time to VTE occurrence (≥ 3 vs. < 3, log-rank test, *P* = 0.078). The patients with KS ≥ 3 (high-risk) had a VTE incidence of 30%, while the ones with KS = 1–2 (intermediate risk) had a disease incidence of 10%, which is somewhat comparable to the results of a previous study among patients with gynaecological tumours^45^. Also following the literature, the original cut-off score (≥ 3) seems to be more appropriate to determine VTE risk among these patients^46^. Although out of the scope of this study, KS ≥ 3 was found to predict a worse prognosis (≥ 3 vs. < 3, log-rank test, *P* = 0.046). Overall, data on the discriminatory performance of KS among cancer patients is inconsistent^20,47,48^. Further investigation is, however, required to validate these findings among CC patients, particularly in larger cohorts and considering other treatments.

As for the polymorphisms, starting with rs10747514, this intronic variant located in *PROCR* leads to the substitution of a guanine (G) for an adenine (A)^49^. The gene *PROCR* located in chromosome 20 encodes for the human endothelial protein C receptor (EPCR), a transmembrane glycoprotein predominantly present in the membrane of endothelial cells, being engaged in haemostasis^50–53^. Specifically, this protein is implicated in the protein C (PC) anticoagulant pathway by acting as the cellular receptor of PC, a serine protease with anticoagulant activity^52–54^. During haemostasis triggering, circulating PC binds to EPCR, leading to the generation of APC. Once dissociated from EPCR, APC together with its cofactor protein S (PS) inhibits the activated coagulation factor V (FVa) and the activated coagulation factor VIII (FVIIIa), consequently downregulating thrombin generation and preventing venous thrombogenesis^53,55^. As for *PROCR* rs10747514, its functional consequence is unclear. Indeed, intronic variants may affect either gene expression, for instance by disrupting a transcription factor binding site or changing the protein function [196, 197]. Thus, it is plausible that *PROCR* rs10747514 might affect EPCR expression and/or the protein activity with further implications in VTE development. In this study, the *PROCR* rs10747514 AA genotype was associated with a lower time to VTE occurrence compared to the G allele, suggesting that the AA genotype has a prothrombotic effect among CC patients (log-rank test, *P* = 0.021). This effect was corroborated by both univariate (AA vs. AG/GG; HR 3.463; 95% CI 1.128–10.369; *P* = 0.030) and multivariate Cox regression analysis adjusted for FIGO stage (AA vs. AG/GG; aHR 4.273; 95% CI 1.342–13.601; *P* = 0.014). Namely, patients carrying the AA genotype had a fourfold increase in the 10-year risk of VTE compared to their counterparts. Together with the literature, it was hypothesized that the AA genotype might either lead to lower EPCR levels and/or activity or to a higher shedding of the receptor from the endothelial membrane (an effect reported for another *PROCR* polymorphism), thus impairing EPCR anticoagulant effect^56,57^.

Beyond EPCR’s implication in the haemostatic pathway, this protein has also been suggested to interfere with carcinogenesis^58,59^. Indeed, EPCR through coagulation-independent mechanisms plays an important role in several tumorigenic-related processes, including apoptosis, angiogenesis, inflammation, cell migration and proliferation^52,55,60^. For instance, the interaction of APC with EPCR and protease-activated receptors (PAR) allows the upregulation of the antiapoptotic protein B-cell lymphoma 2 (BCL-2), as well as the downregulation of p53, Bax and caspases 3, 8 and 9, therefore promoting cell survival^55^. Altogether, growing evidence strongly suggests a tumour-promoting effect of EPCR^52,55,58,60–62^. Concordantly, in this study, *PROCR* rs10747514 was found to have a prognostic value among CC patients, with the AA genotype exhibiting a detrimental impact on the 10-year OS of the patients (AA vs. AG/GG; log-rank test, *P* = 0.040). When stratifying the analysis according to VTE status, a significant prognostic impact of the *PROCR* rs10747514 was only observed among the group of patients without clinical evidence of VTE occurrence (AA vs. AG/GG; log-rank test, *P* = 0.004). The most likely explanation for the absence of a prognostic value in the context of VTE is the reduced number of VTE patients with available information concerning the polymorphism genotypes (n = 17), which may have prevented the detection of a significant association (AA vs. AG/GG; log-rank test, *P* = 0.087). Nevertheless, considering solely the patients without clinical evidence of VTE, the *PROCR* rs10747514 AA genotype was associated with a lower 10-year OS compared to the G allele genotypes in the group of younger (AA vs. AG/GG; log-rank test, *P* = 0.005) and early disease stage CC patients (AA vs. AG/GG; log-rank test, *P* = 0.003), confirming the negative impact of the AA genotype. Indeed, the patient’s age and cancer stage are well-known factors related to thrombogenesis^1^. Specifically, age combined with sex hormonal changes is reported to influence the expression levels of proteins involved in the coagulation system^63^. Namely, activation of the PC pathway is suppressed with ageing, which is thought to be one of the mechanisms underlying age-associated thrombosis^64^. Therefore, assuming that younger patients have a higher *PROCR* expression, the AA genotype might impose an additive effect by increasing EPCR activity, consequently favouring cancer pathways. As for the cancer stage, most genetic polymorphisms associated with thrombogenesis seem to be only relevant before metastasis. These variants might contribute to metastatic dissemination, but once it happens other biological mechanisms might be more preponderant in cancer progression^4^. Univariate (AA vs. AG/GG; HR 2.578; 95% CI 1.308–5.083; *P* = 0.006) and multivariate Cox regression analysis adjusted for FIGO stage (AA vs. AG/GG; aHR 2.612; 95% CI 1.322–5.161; *P* = 0.006) confirmed the prognostic value of *PROCR* rs10747514 in the cohort of CC patients without clinical evidence of VTE. Namely, patients carrying the AA genotype had a threefold increase in 10-year risk of death compared to their counterparts in both analyses. Altogether, it was hypothesized that the AA genotype might be associated with higher EPCR levels and/or activity, thereby promoting tumorigenesis and deteriorating the CC patients’ clinical outcome. However, the prothrombotic effect of the AA genotype observed in this study (AA vs. AG/GG; aHR 4.273; 95% CI 1.342–13.601; *P* = 0.014) seems to oppose this hypothesis. Although future functional studies are required to elucidate the underlying biological processes, one possible explanation could be an increased EPCR expression and/or activity paralleling with PC trapping by EPCR, thus impairing the anticoagulation pathway while promoting cancer progression through coagulation-independent mechanisms in individuals without symptomatic VTE. Overall, *PROCR* rs10747514 may be a useful biomarker to predict VTE among CC patients and assess their prognosis even in the absence of venous thrombogenesis.

The intronic variant rs2502448 located in *RGS7* results in a substitution of a thymine (T) to a cytosine (C). The gene *RGS7* is located in chromosome 1 and encodes for the Regulator of G-protein signalling 7 (RGS7)^49^. This protein belongs to the multifunctional superfamily of regulators of G-protein signalling (RGS) responsible for controlling the signalling cascades of G-protein, an enzyme engaged in cell signalling transduction. The G-protein activity is actively controlled by RGSs (including RGS7) and G protein-coupled receptors (GPCRs). While the former inhibits G-protein signalling through the hydrolysis of guanosine triphosphate (GTP), the latter stimulates GTP-binding leading to G-protein activation^65–68^. Among its several functions, RGS7 has been previously described to play a role in platelet function, with some genetic polymorphisms within *RGS7* demonstrated to be associated with a risk for venous thrombosis and ischemic stroke^66,67,69–71^. As GPCRs are crucial for platelet activity, RGS7, an antagonist of GPCR signalling transduction, might prevent platelet aggregation, consequently reducing the risk of venous thrombogenesis^65,68,72^. Although its functional consequence is unclear, as an intronic variant, *RGS7* rs2502448 might alter RGS7 expression and/or protein functionality, favouring VTE development. Thus, considering that the *RGS7* rs2502448 C allele was previously associated with higher platelet reactivity under acetylsalicylic acid (ASA; also known as aspirin) treatment (odds ratio (OR) 3.45; CI 1.82–6.53), and given the potential RGS7 negative regulation of platelet activation and aggregation, the C allele might be linked to lower protein expression and/or activity, favouring venous thrombogenesis^36,71^. Concordantly, in this study, the *RGS7* rs2502448 C allele was associated with a lower time to VTE, indicating a prothrombotic effect of this allele among CC patients (log-rank test, *P* = 0.006). This observation was further confirmed by both univariate (CT/CC vs. TT; HR 10.062; 95% CI 1.329–76.179; *P* = 0.025) and multivariate Cox regression analysis adjusted for FIGO stage (CT/CC vs. TT; aHR 10.672; 95% CI 1.409–80.813; *P* = 0.022). Specifically, patients carrying the C allele genotypes had a tenfold increase in the 10-year risk of VTE compared to their counterparts.

Beyond haemostasis, RGS play several roles, many of those involved in malignancy. RGS expression levels have been reported to be deregulated in a tissue-specific manner, particularly RGS1, mediating cellular processes such as survival, proliferation, and migration^73–76^. Inclusively, genetic variants in RGS genes have been found to have a prognostic value among oncological patients^76,77^. Little is known, however, about the role of RGS7 in tumorigenesis, particularly in the context of CC since RGS most likely acts in a tissue-specific manner^76^. In the present study, *RGS7* rs2502448 was marginally associated with the 10-year OS of the CC patients, with the C allele exhibiting a benefit impact (CT/CC vs. TT; log-rank test, *P* = 0.086). Based on the previously mentioned hypothesis that the C allele might be linked to lower RGS7 expression and/or activity, thereby favouring venous thrombogenesis, RGS7 may exert oncogenic roles, which would explain the beneficial impact associated with the *RGS7* rs2502448 C allele. Nevertheless, additional studies are required to explore the role of RGS7 in cervical tumorigenesis. Like *PROCR* rs10747514, a significant impact of *RGS7* rs2502448 on patients’ 10-year OS was only observed among the group of patients without clinical evidence of VTE (CT/CC vs. TT; log-rank test, *P* = 0.010). Among these patients, the *RGS7* rs2502448 C allele was associated with a higher 10-year OS compared to the TT genotypes in the group of younger (CT/CC vs. TT; log-rank test, *P* = 0.019) and early disease stage CC patients (CT/CC vs. TT; log-rank test, *P* = 0.005), corroborating the beneficial impact of C allele. Indeed, age-associated changes in the expression level of RGS proteins have been reported^78,79^. Thus, it was hypothesized that younger patients may have a higher expression of RGS7, meaning that in these patients the polymorphism rs2502448 may have an additive impact. As for the influence of the cancer stage, as previously mentioned, genetic polymorphisms associated with thrombogenesis are thought to be more impacting in a premetastatic context, favouring events towards metastasis. Concordantly, *RGS7* rs2502448 was previously found to be associated with the 5-year OS of epithelial ovarian cancer (EOC) patients at early disease stages (log-rank test, *P* = 0.035) but not at advanced stages, suggesting a potential role of this variant in metastasis regardless of tumour type^36^. The prognostic value of *RGS7* rs2502448 in patients without clinical evidence of VTE was corroborated by both univariate (CT/CC vs. TT; HR 0.513; 95% CI 0.305–0.862; *P* = 0.012) and multivariate Cox regression analysis adjusted for FIGO stage (CT/CC vs. TT; aHR 0.490; 95% CI 0.288–0.835; *P* = 0.009). Namely, patients carrying the C allele had a 50% reduction in the 10-year risk of death compared to their counterparts in both analyses. Altogether, it was hypothesized that the C allele may lead to lower expression levels of RGS7, thus having a beneficial effect on CC patients’ clinical outcomes, assuming that the protein has oncogenic roles. Our research group previously found that, among EOC patients, the rs2502448 C allele was also associated with a higher 5-year OS compared to the TT genotype (log-rank test, *P* = 0.035), also leading to the hypothesis of lower protein levels associated with the C allele^36^. Overall, like *PROCR* rs10747514, *RGS7* rs2502448 seems to be a valuable tool to predict VTE among CC patients and assess their prognosis even in the absence of the disease.

The remaining evaluated polymorphisms were not associated with VTE development or the patient’s prognosis (*P* > 0.05). Nevertheless, as this is a preliminary study, additional investigation with a larger cohort is required to validate this study’s findings. Briefly, the intronic variant rs4734879 located within *ZFPM2* is thought to be associated with the expression levels of *Vascular endothelial growth factor A* (*VEGFA*) and other haemostatic components implicated in thrombogenesis^49,80–83^. Overall, in the context of cancer-related VTE, this variant has not been validated yet, at least to the best of our knowledge^4^. In a previous study conducted by our research group, *ZFPM2* rs4734879 was found to be a prognostic factor among EOC patients, which was attributed to its potential influence in tumour neo-angiogenesis^35^. The synonymous variant rs7164569 located in *OTUD7A* might be associated with the downregulation of Nuclear Factor Kappa B (NF-kB), which positively regulates platelet activity^49,84^. Since OTUD7A acts towards NF-kB inhibition, this deubiquitinase might impair platelet responses decreasing the risk of thrombogenesis^85–89^. However, no study has yet validated the role of rs7164569 in cancer-related VTE^4^. As the NF-kB signalling pathway is implicated in several processes associated with tumorigenesis, OTUD7A by inhibiting it may suppress these cellular processes, suggesting that this deubiquitinase may impose a protective effect in cancer progression^87,88^. To the best of our knowledge, however, the variant rs7164569 has not yet been linked to the prognosis of oncological patients^35^. The missense variant *ITGB3* rs5918 has been extensively studied given its putative impact on platelet responses and venous thrombogenesis in the general population^37,49,90^. Until now, however, no study has yet demonstrated the role of this polymorphism in cancer-related VTE^4^. Beyond platelet activation, which itself promotes cancer progression since platelets are engaged in many processes towards tumour dissemination, GP IIb/IIIa is implicated in several signalling pathways involved in tumour progression. Concordantly, the variant showed a prognostic value among EOC patients^36^. The intronic variant *GSR* rs3779647 was previously associated with platelet activation, however, to the best of our knowledge, this genetic variant has not been linked to cancer-related VTE^4,49,71,91^. The polymorphism was previously associated with EOC patients’ survival, which is thought to be due to the contribution of GSR to resistance to platinum-based chemotherapy through its role in glutathione metabolism^36,92^. The intronic variant rs4253417 locates within the *F11* gene that encodes for the coagulation factor XI (FXI), which is implicated in the intrinsic coagulation pathway culminating with thrombin generation and consequently fibrin deposition^49,93,94^. The variant rs4253417 was found to be associated with FXI circulating levels but has not been validated in the context of malignancy^4,95^. No direct role of FXI in tumorigenesis is known, however, it may indirectly influence cancer pathways by modulating the activity and/or levels of downstream coagulation proteins^96–105^. The intergenic variant rs6764623 locates close to *CNTN6,* which encodes for a protein that seems to trigger the Notch pathway, which mediates inflammatory responses implicated in cardiovascular disorders^49,106^. Although unclear, the polymorphism is thought to regulate gene expression through cis and trans effects, potentially influencing CNTN6 levels^35^. The variant has been associated with a thrombotic risk in the general population, however, the same has not yet been demonstrated among oncological patients^4,85^. Also, rs6764623 was shown to have a prognostic value among EOC patients, which could be due to the multiple roles of the Notch signalling pathway in carcinogenesis^35^.

The most notable limitation of the study was its retrospective nature, which prevented the collection of relevant data. Specifically, as it is well known, several factors modulate the risk for VTE development, including, but not limited to, other comorbidities (for instance, haematological diseases), pregnancy, breastfeeding, use of oral contraception and a history of VTE, which were not accounted for in this study due to lack of information for most patients^1^. Furthermore, data concerning the use of anticoagulants or antiaggregating agents prior to cancer diagnosis were also unavailable and given that no active screening of VTE is included in the clinical routine procedures, asymptomatic VTE events are not accounted for, which could partially explain the poor performance of KS. Inclusively, as VTE was not actively screened, the distribution of patients with and without VTE could be different, which represents an intrinsic limitation in this study. Another important data missing was the specific cause of death among these patients, which prevented the assessment of cancer specific survival, as well as a possible VTE-related mortality. Likewise, information on the discontinuation of antineoplastic treatment due to VTE, which may impact the patients’ survival, is also missing. Lastly, the temporal gap regarding the patients’ recruitment (February 2002 to October 2009 and May 2017 to October 2021) due to the requirement of biological samples may have led to selection bias.

## Conclusion and future perspectives

Even when there is a successful antithrombotic treatment, VTE is associated with a spectrum of complications that deteriorate cancer patients’ prognosis^107^. Thus, the primary effort of investigation on cancer-related VTE should be focusing on the disease prevention. Altogether, the results of the present study corroborate the detrimental effect VTE has on the survival of CC patients and suggest that the genetic variants *PROCR* rs10747514 and *RGS7* rs2502448 could be useful predictive biomarkers of thrombogenesis in these patients, in addition to serving as potential tools for prognosis assessment regardless of VTE status. In opposition, the KS was only marginally associated with CC-related VTE, showcasing a poor performance. Overall, the present study highlights the need for a more tailored thromboprophylaxis among oncological patients and better and more personalized cancer management considering the role of haemostatic components in tumorigenesis. In this sense, thrombogenesis-associated genetic polymorphisms might be the bridge to connect both worlds. However, as this is a preliminary study with limitations, additional investigation in larger cohorts and considering other relevant factors are required to validate the results. Prospective studies with active screening of VTE are also a must to better explore the role of *PROCR* rs10747514 and *RGS7* rs2502448 and assess the performance of KS among CC patients. Likewise, it would be relevant to compare the predictive ability of the thrombogenesis-related polymorphisms with the KS and investigate potential drug targets that could be used for both thromboprophylaxis and CC treatment.
